# Apoptosis induction by oxidized glycated LDL in human retinal capillary pericytes is independent of activation of MAPK signaling pathways

**Published:** 2009-01-19

**Authors:** J. Matthew Diffley, Mingyuan Wu, Mimi Sohn, Weiwei Song, Samar M. Hammad, Timothy J. Lyons

**Affiliations:** 1 Harold Hamm Oklahoma Diabetes Center and Section of Endocrinology and Diabetes, University of Oklahoma Health Sciences Center, Oklahoma City, OK; 2 Division of Neuroscience, Medical University of South Carolina, Charleston, SC; 3 Department of Cell Biology and Anatomy, Medical University of South Carolina, Charleston, SC

## Abstract

**Background:**

Pericyte loss is a cardinal feature of early diabetic retinopathy. We previously reported that highly oxidized-glycated low density lipoprotein (HOG-LDL) induces pericyte apoptosis in vitro. In this study, we investigated the role of the mitogen-activated protein kinase (MAPK) signaling pathways in HOG-LDL-induced apoptosis in human pericytes.

**Methods:**

Human retinal capillary pericytes (HRCP) were exposed to native LDL (N-LDL) and HOG-LDL, and apoptosis was measured using flow cytometry. Time- and dose-dependent responses of extracellular signal-regulated kinase (ERK), p38, and Jun N-terminal kinase (JNK) following exposure to N-LDL or HOG-LDL were determined using western blotting. U0126 (ERK inhibitor), SB203580 (p38 inhibitor), and SP600125 (JNK inhibitor) were used to determine the role of MAPK signaling in HOG-LDL-induced apoptosis.

**Results:**

HOG-LDL induced apoptosis in HRCP in a dose-dependent manner at concentrations from 5 to 50 mg/l, with a constant effect from 50 to 200 mg/l. When compared to serum-free medium (SFM), this effect of HOG-LDL was found to be significant at all doses above 10 mg/l. In contrast, N-LDL at 200 mg/l did not induce apoptosis compared with SFM. Exposure to N-LDL versus HOG-LDL induced similar phosphorylation of ERK, p38, and JNK, peaking at 5 min, with similar dose-dependent responses up to 25 mg/l that were constant from 25 to 100 mg/l. Blocking of the ERK, p38, and JNK pathways did not inhibit pericyte apoptosis induced by HOG-LDL.

**Conclusions:**

Our data suggest that apoptosis induced by HOG-LDL in HRCP is independent of the activation of MAPK signaling pathways.

## Introduction

Diabetic retinopathy (DR) is a leading cause of the blindness in the working age (18–65 years old) population [1,2]. An important characteristic of its early stages is pericyte loss [3], which is associated with increased pericyte apoptosis [2,4,5]. Retinal capillary pericytes mediate vascular stability and control endothelial cell proliferation. Loss of pericytes results in endothelial cell proliferation and enhances abnormal angiogenesis in the retina [5]. Although the pathogenesis of pericyte loss is not clear, poor glycemic control, hypertension, dyslipidemia (especially modified low-density lipoproteins), and duration of diabetes are all implicated [6-9]. It is hypothesized that retinal capillary leakage during the early stage of DR [10] enables LDL to be extravasated and trapped in the extravascular and subendothelial spaces, and that subsequent glycation and oxidation of extravasated LDL under hyperglycemia and enhanced oxidative stress lead to retinal vascular injury [11-13]. These notions are supported by our previous studies, which showed highly oxidized-glycated low density lipoprotein (HOG-LDL) significantly induced apoptosis in cultured bovine retinal capillary endothelial cells and pericytes, and in human retinal capillary pericytes (HRCP) [14-16], and induced many alterations in gene expression and function in HRCP [17,18]. Further, our recent immunohistochemical studies have shown that oxidized LDL is absent in healthy retinas, but present in diabetic retinas to an extent proportional to the severity of DR [16]. The underlying mechanisms by which HOG-LDL may trigger pericyte loss include induction of DNA fragmentation, activation of caspase pathways, and mitochondrial dysfunction [15,16].

The mitogen-activated protein kinase (MAPK) signaling pathways are activated by different extracellular stimuli, resulting in a wide range of cellular responses, including apoptosis, proliferation, and inflammation. In mammals, three major MAPK pathways have been identified: extracellular signal-regulated kinases (ERK), p38, and stress activated protein kinases (SAPK)/c-Jun-N-terminal kinase (JNK). Accumulating studies show that MAPK pathways are associated with apoptosis triggered by oxidized LDL in vascular cells [19-21]. With regard to pericyte loss, phosphorylation of p38 MAPK is involved in retinal capillary pericyte loss induced by modification of fibronectin with alpha-dicarbonyl compounds [22]. Therefore, the possibility that MAPK signaling pathways might be involved in pericyte loss induced by modified LDL merits investigation.

In the present study, we investigated whether the apoptotic effects of HOG-LDL versus native LDL (N-LDL) on HRCP are associated with alterations in the activation of MAPK signaling pathways. We tested the involvement of the three known series of MAPK cascades: ERK1/2, p38, and JNK. The results showed that N-LDL and HOG-LDL activated all three MAPK signals, but to a similar extent, and that inhibition of the ERK, p38, and JNK pathways did not affect the amount of apoptosis induced by HOG-LDL. Therefore, we conclude that apoptosis induced by exposure to HOG-LDL in HRCP is independent of activation of MAPK pathways.

## Methods

This study was approved by the Institutional Review Boards at the University of Oklahoma Health Sciences Center (Oklahoma City, OK) and the Medical University of South Carolina (Charleston, SC). It was conducted according to Declaration of Helsinki principles, with written informed consent obtained from all study participants

### Cell culture

HRCP were obtained from Cambrex (Walkersville, MD). These cells were cultured with EBM-2 basal serum-free culture medium (SFM) and EGM-2-MV SingleQuots growth supplement, which contained the following ingredients obtained from Clonetics^®^ (Walkersville, MD): 5% fetal bovine serum, 0.1% hEGF, 0.04% hydrocortisone, 0.1% VEGF, 0.4% hFGF-B, 0.1% R^3^-IGF-1, 0.1% ascorbic acid, and 0.1% GA-1000. HRCP from passages 4–10 were used.

### LDL isolation and modification

Protocols employed were as previously reported [17,23]. Briefly, human LDL was isolated by sequential ultracentrifugation (d=1.019–1.063) of pooled plasma. The pooled plasma was obtained on seven occasions, and was provided on each occasion by 4–6 fasting normal healthy volunteer subjects who were recruited by advertising. Different groups of volunteer subjects were used on each occasion to ensure that results that could be generalized. Volunteers, 71% of whom were male, were aged 20 to 40 years, and were taking neither prescription medications nor antioxidant vitamins. Each volunteer was documented as being non-diabetic (normal HBA_1_c, fasting glucose), having normal renal function (blood urea nitrogen, serum creatinine), and normal serum lipid profiles. N-LDL and glycated LDL (G-LDL) were prepared by incubating LDL with and without freshly prepared 50 mM glucose for 72 h at 37 °C under anti-oxidant conditions (1 mM N,N-bis[2-(bis[carboxymethyl]-amino)ethyl]glycine (DTPA) and 270 μM EDTA, with sustained nitrogen gas). HOG-LDL was prepared from G-LDL by oxidizing in the presence of 10 μM CuCl_2_ (24 h, 37 °C), followed by dialysis at 4 °C for 24 h. Protein in LDL preparations was determined by BCA protein assay (Pierce, Rockford, IL). LDL preparations were further characterized by measuring fluorescence at 360 nm ex/430 nm em (Gilford Fluorimeter IV, Oberlin, OH), performing agarose gel electrophoresis (Paragon® LIPO Gel, Beckman, Fullerton, CA), and measuring absorbance at 234 nm using a Beckman DU 650 spectrophotometer. LDL preparations were stored in the dark under nitrogen at 4 °C in the presence of 270 μM EDTA, and were used within six weeks. Experiments were repeated using different human samples for each pooled LDL preparation.

### Apoptosis: time-dependent and dose-dependent responses

HRCP were grown to 90% confluence in T25 flasks (Cellstar®, Greiner Bio-One, Frickenhausen, Germany), then rendered quiescent by 24 h exposure to SFM. HRCP were then treated with SFM, 200 mg/l N-LDL, or 5–200 mg/l of HOG-LDL at 37 °C for 12 h. A combination of N-LDL and 100 or 200 mg/l HOG-LDL for 12 h was administered to investigate whether N-LDL protects HRCP from the toxic effects of HOG-LDL. Cells were harvested using trypsin, washed with PBS (8 g NaCl, 2 g KCl, 2.16 g Na_2_HPO_4,_ 7 g H_2_O, 0.208 g KH_2_PO_4_ per liter, MilliQ water, pH 7.36–7.44), and incubated with Annexin V-FITC and propidium iodide (PI) for 10 min The apoptotic signals were determined using an ExCalibur Flow Cytometer (Becton Dickinson, San Jose, CA). For fold difference calculations, percent apoptotic values were normalized against the control treatment.

### MAPK time-response and dose–response assays

Cells were grown to 90% confluence in 6 well tissue culture plates (Costar^®^, Corning, NY) or 60 mm tissue culture dishes (Becton Dickinson, Franklin Lakes, NJ) and were rendered quiescent by 24 h exposure to SFM. For the time-response assays, the cells were exposed to either 100 mg/l N-LDL or 100 mg/l HOG-LDL in SFM from 1 to 60 min. For the dose–response assays, the cells were exposed to 1–100 mg/l N-LDL or HOG- LDL for 5 min. Cells were harvested for measurement of MAPK protein by western blot.

### Western blotting

Cell layers were washed three times with ice-cold PBS, and then exposed to a detergent buffer, which consisted of 1%Triton X-100, 0.5% Tween-20, 0.5 M NaCl, and 50 mM Hepes, pH 7.5, and also contained a protease inhibitor mixture (EDTA-free; Roche, Mannheim, Germany). Cells were scraped from the dish with a rubber policeman and lysed with a 28 gauge needle. Protein concentrations in extracts were determined by BCA protein assay. Equal amounts of protein (15 μg) were separated on Novex® pre-cast gels, which contained Tris-glycine, 4%–20% polyacrylamide gradient (Invitrogen, Carlsbad, CA), under reducing conditions, then transferred to fluoride membranes (Pall, Ann Arbor, MI). The membranes were blocked in 5% nonfat dry milk in Tris-buffered saline (TBS), which contained 50 mM Tris, and 150 mM NaCl, pH 7.4. The blocked membranes were immunoblotted with a 1:1,000 dilution rabbit polyclonal tyrosine phospho-specific ERK1/2, p38 MAPK, or SAPK/JNK antibodies (Cell Signaling Technology, Beverly, MA), diluted in TBS containing 5% dry nonfat dry milk (for ERK1/2) or 5% BSA (for p38 and SAPK/JNK) and 0.1% Tween-20, overnight at 4 °C. Membranes were then washed with TBS 0.1% Tween-20. Total MAPK was measured in the same membranes by stripping the membranes with 100 mM glycine, pH 2.3, for 5 min and immunoblotting with a 1:1,000 dilution total ERK1/2, p38 MAPK, and SAPK/JNK antibodies (Cell Signaling Technology). The membranes were incubated with a secondary antibody conjugated to horseradish peroxidase (Amersham, Piscataway, NJ). Immunoreactive bands were visualized using the chemiluminescence reagent ECL plus™ (Amersham Pharmacia, Piscataway, NJ). The bands for phosphorylated and total forms were detected at sites corresponding to known molecular weights in all immunoblots. Each band for P-ERK, P-p38, or P-JNK was quantified by densitometry and normalized to T-ERK, T-p38, or T-JNK level using the Scion Image program (Frederick, MD). After this analysis, for purposes of presentation in Figures, the blot images were truncated to remove portions of lanes that were devoid of signal.

### Apoptosis in the presence of MAPK inhibitors

To determine effects of MAPK inhibitors on apoptosis and cytotoxicity, we preinubated cells with each of the following inhibitors for 1 h: 10 µM U0126 (ERK inhibitor), 10 µM SB203580 (p38 inhibitor), and 10 µM SP600125 (JNK inhibitor). LDL was then added to the medium, and cells were incubated for a further 1 h. Medium was changed to SFM, cells were incubated for a further 12 h, then harvested. This modification of the aforementioned protocol (cells were only exposed to LDL for 1 h) still yielded statistically significant differences between 200 mg/l N-LDL and 200 mg/l HOG-LDL without risking the degradation of the inhibitors that might have occurred over the 12 h period.

### Statistical analysis

Results were expressed as mean±SD, and statistical significance was assessed by paired two-tailed Student’s *t*-test. Squares regression was used to test dose dependence. The level of significance was set at p<0.05.

## Results

### LDL-induced apoptosis

**Figure 1:**
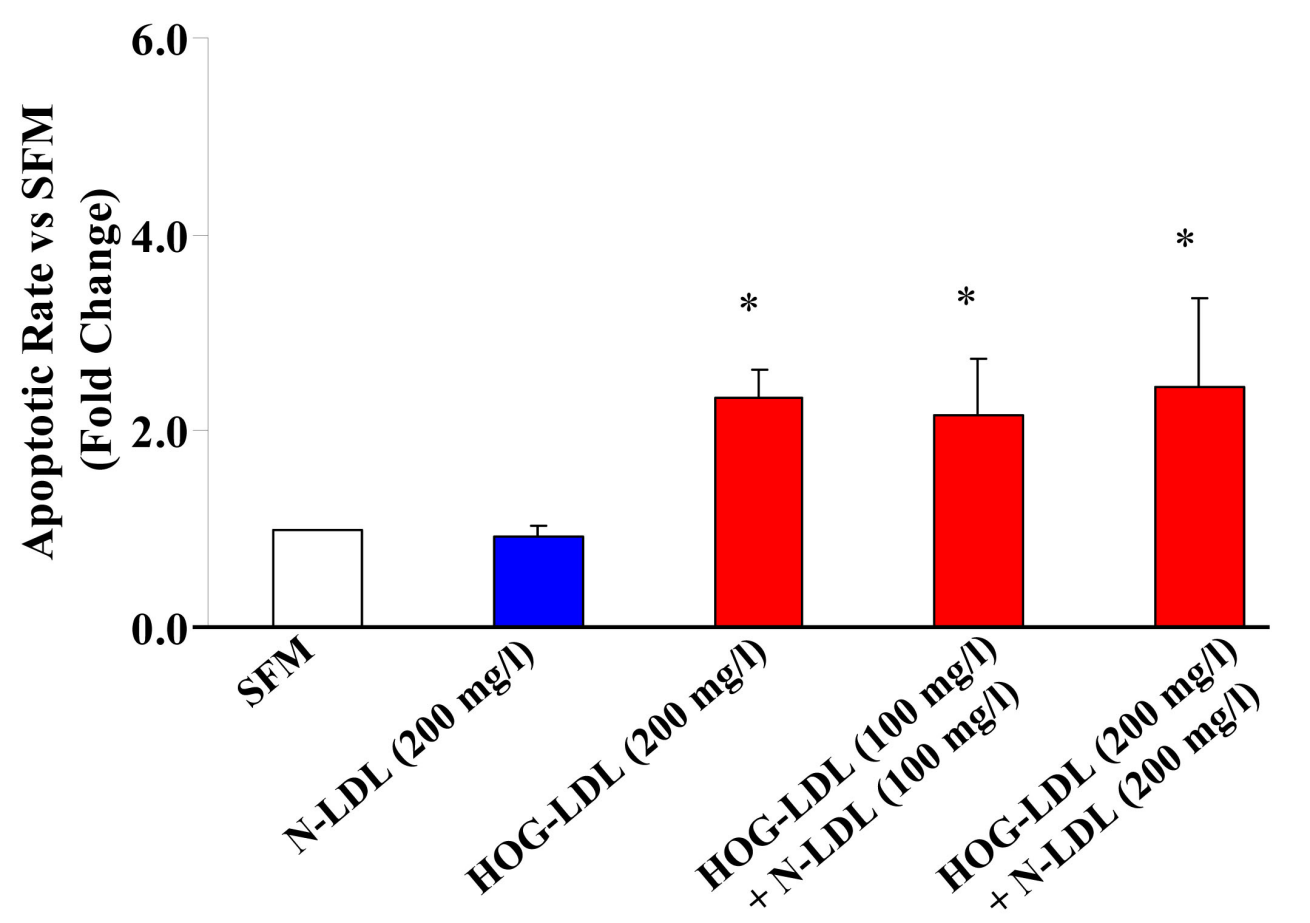
HOG-LDL induces apoptosis in HRCP. After 12 h, 200 mg/l highly oxidized-glycated low density lipoprotein (HOG-LDL) caused a 2–3 fold increase in apoptosis (measured by Annexin V FITC and propidium iodide) compared to serum-free medium (SFM) and native-low density lipoprotein (N-LDL). A combination of N-LDL and HOG-LDL did not inhibit HOG-LDL-induced apoptosis. Bars represent mean±SD of three separate experiments. The asterisk indicates p<0.05 compared to SFM.

**Figure 2:**
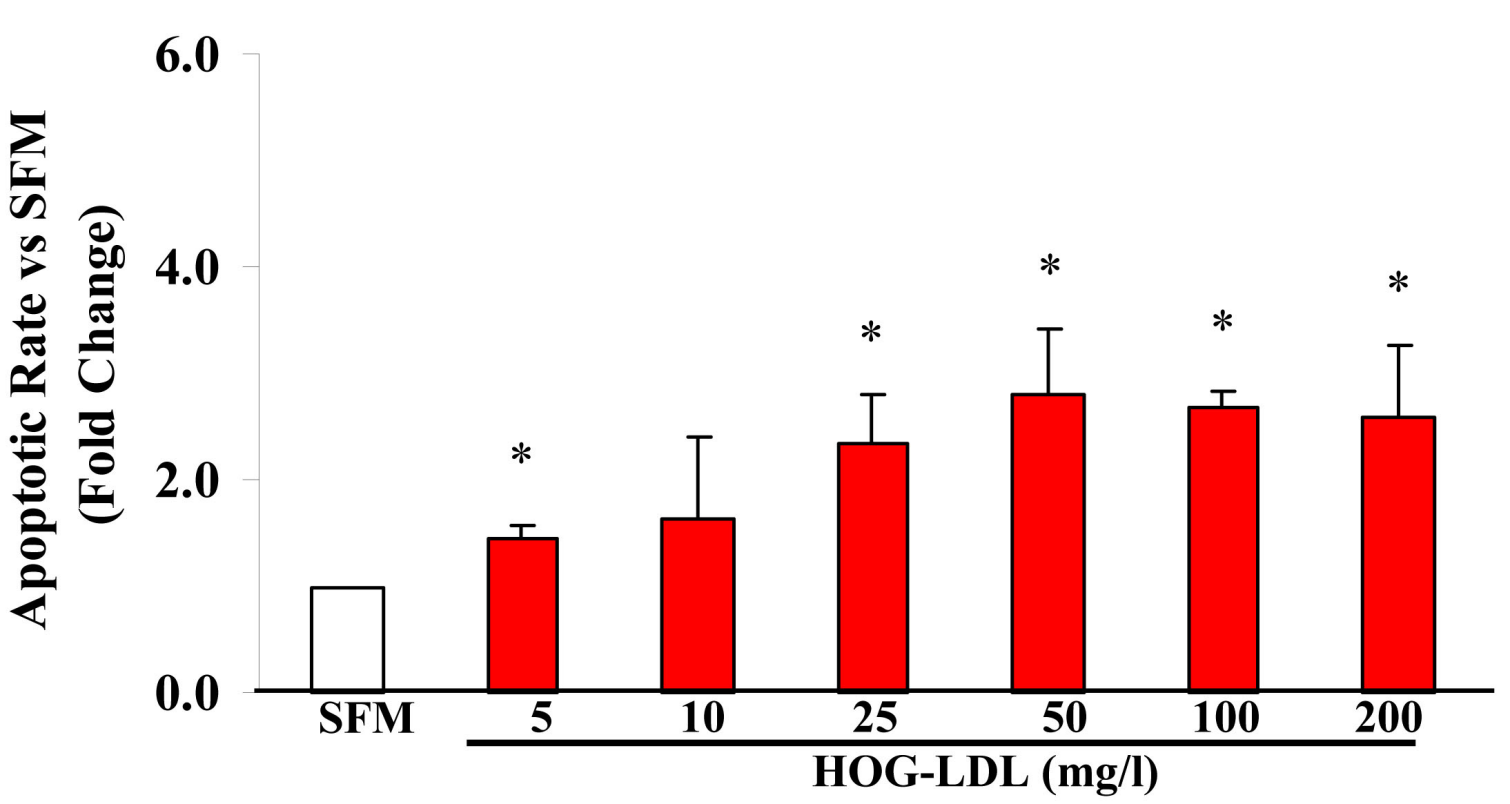
Effects of HOG-LDL on apoptosis of human retinal capillary pericytes. Highly oxidized-glycated low density lipoprotein (HOG-LDL) at all doses, except 10 mg/l, significantly increased apoptotic rate compared to serum-free medium (SFM). The asterisk indicates p<0.05 compared to SFM; n=3. Squares regression also showed a dose-dependent effect of HOG-LDL (below 50 mg/l) on survival of human retinal capillary pericytes.

HRCP exposed to HOG-LDL (200 mg/l) for 12 h showed a 2- to 3-fold increase in apoptosis versus either SFM or N-LDL (200 mg/l). HRCP exposed to N-LDL (200 mg/l) for 12 h showed no increase in apoptosis versus SFM (Figure 1). A combination of N-LDL and HOG-LDL did not mitigate the increased apoptosis induced by HOG-LDL alone (Figure 1). Cells incubated for 12 h with 0, 5, 10, 25, 50, 100, and 200 mg/l of HOG-LDL showed a dose-dependent apoptotic response, maximal at 50 mg/l and constant at the higher doses (Figure 2). At all doses, except 10 mg/l, the increase in apoptotic rate induced by HOG-LDL versus SFM was significant (Figure 2).

### ERK activation

#### N-LDL and HOG-LDL induced ERK1/2 phosphorylation in a time-dependent manner

**Figure 3:**
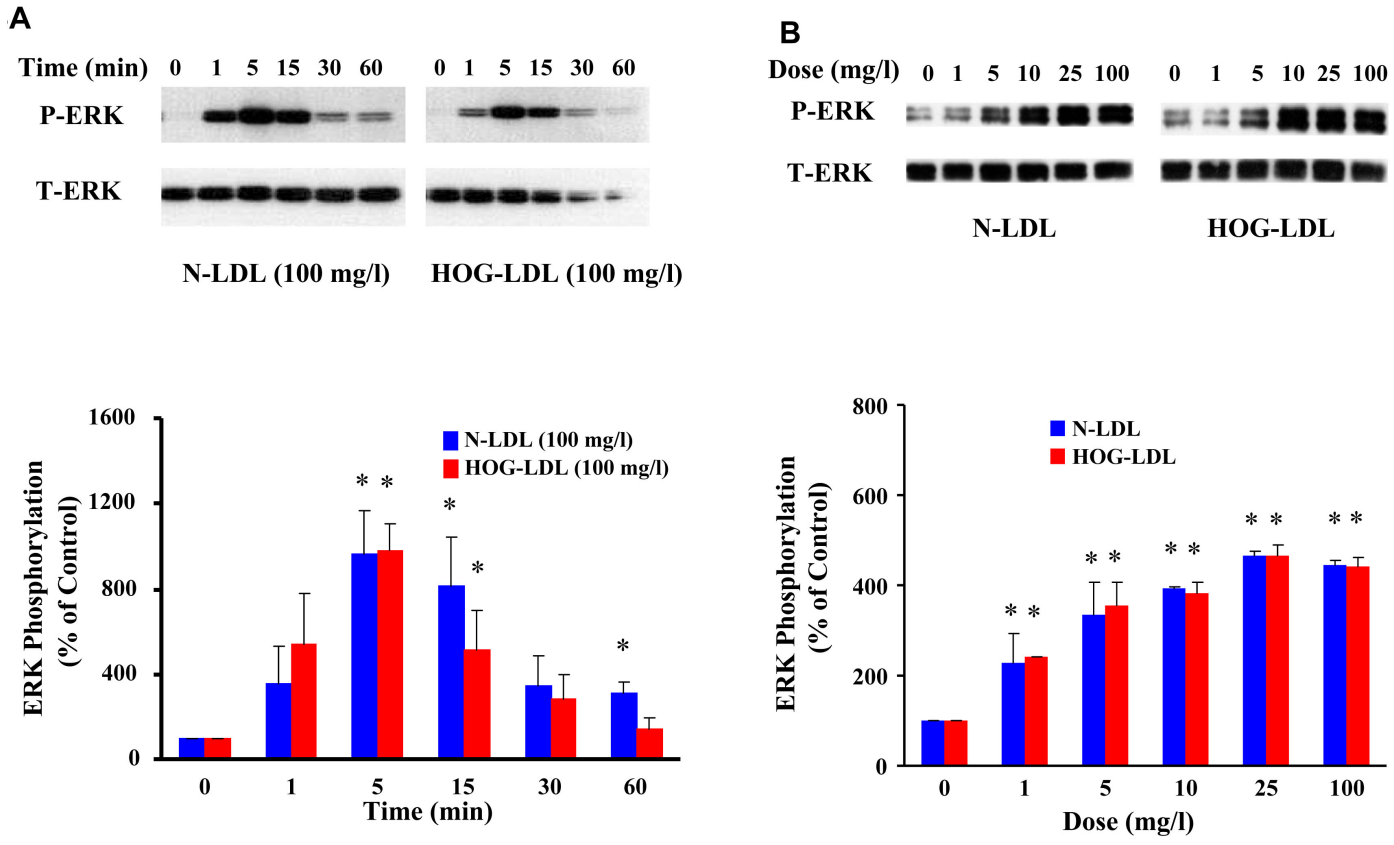
LDL increases ERK phosphorylation. **A:** This panel shows representative western immunoblots from one experiment, and densitometric data from three experiments (mean±SD) describing the time course of LDL-induced ERK phosphorylation (P-ERK and T-ERK means phosphorylated and total ERK). **B:** This panel shows representative western immunoblots from one experiment, and densitometric data from three experiments (mean±SD) describing dose effects of LDL on ERK phosphorylation. N-LDL and HOG-LDL had similar effects on ERK1/2 phosphorylation. Control immunosignal (T-ERK) bands were detected in the same gels as P-ERK after stripping and re-probing. In both panels, densitometric calculations of P-ERK were corrected for T-ERK. Asterisk represents p<0.05 compared to control (Time 0 or Dose 0).

Quiescent HRCP were incubated with 100 mg/l N-LDL or HOG-LDL for 1, 5, 15, 30, and 60 min. For each LDL preparation, ERK1/2 phosphorylation increased at 1 min, peaked at 5 min, and then declined. N-LDL and HOG-LDL induced similar increases in ERK1/2 phosphorylation (Figure 3A).

#### Dose-dependent ERK response at lower concentrations of N-LDL and HOG-LDL

Quiescent HRCP were incubated for 5 min with 1, 5, 10, 25, and 100 mg/l of N-LDL and HOG-LDL. The two preparations induced similar increases of ERK1/2 phosphorylation and exhibited similar dose-dependent curves (Figure 3B).

#### Effect of ERK inhibition on apoptosis

**Figure 4:**
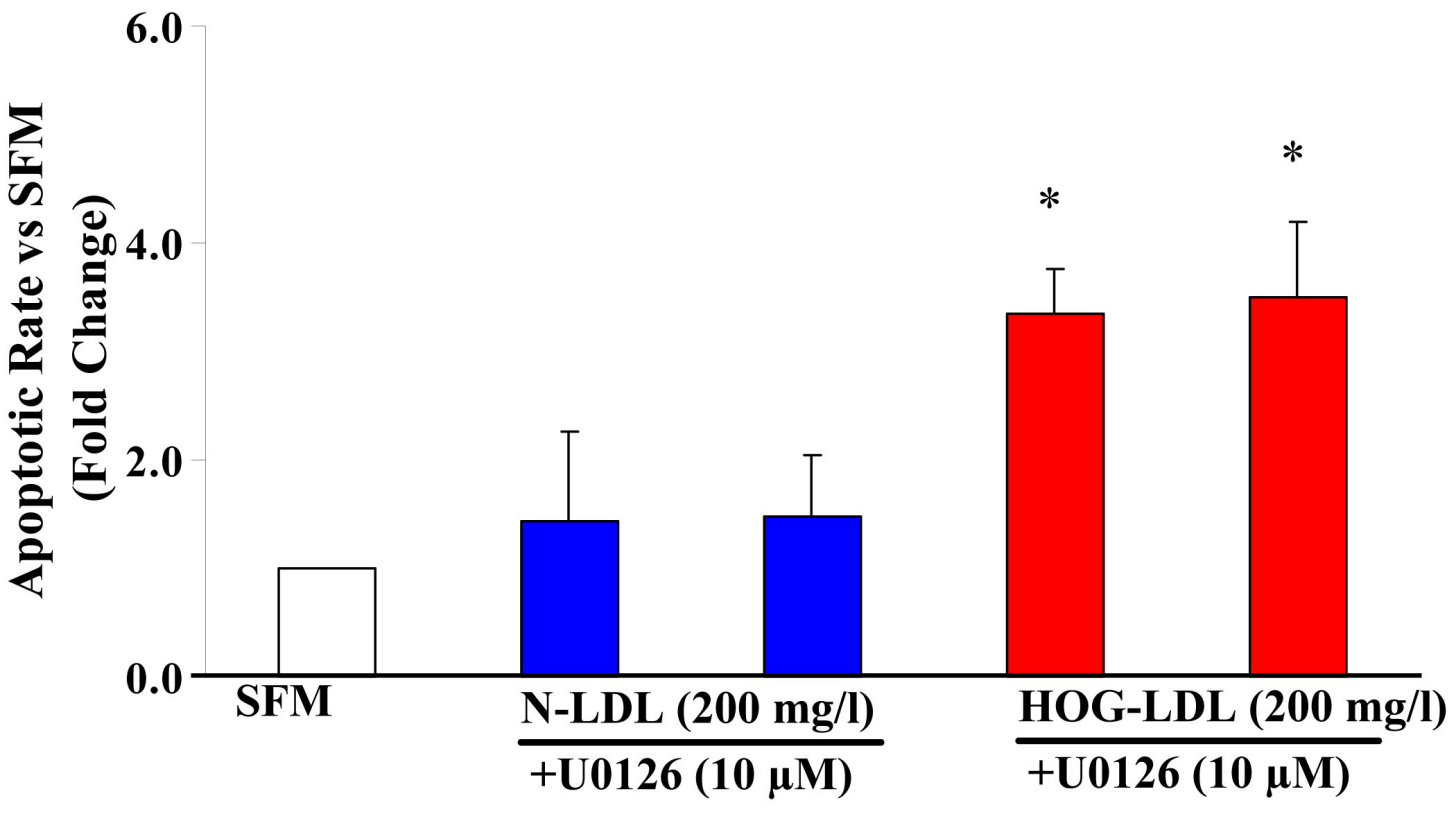
Effects of U0126 on HOG-LDL-induced apoptosis. Administration of 10 µM U0126, an inhibitor of extracellular signal-regulated kinase (ERK) signaling pathways, did not decrease apoptosis induced by at 200 mg/l highly oxidized-glycated low density lipoprotein (HOG-LDL). Bars represent mean±SD of three separate experiments. The asterisk indicates p<0.05 compared to serum-free medium (SFM).

U0126 was employed as an inhibitor of ERK. U0126 (10 µM) did not inhibit apoptosis induced by HOG-LDL at 100 mg/l (data not shown) or at 200 mg/l (Figure 4).

### p38 MAPK activation

#### N-LDL and HOG-LDL induced p38 MAPK phosphorylation in a time-dependent manner

**Figure 5:**
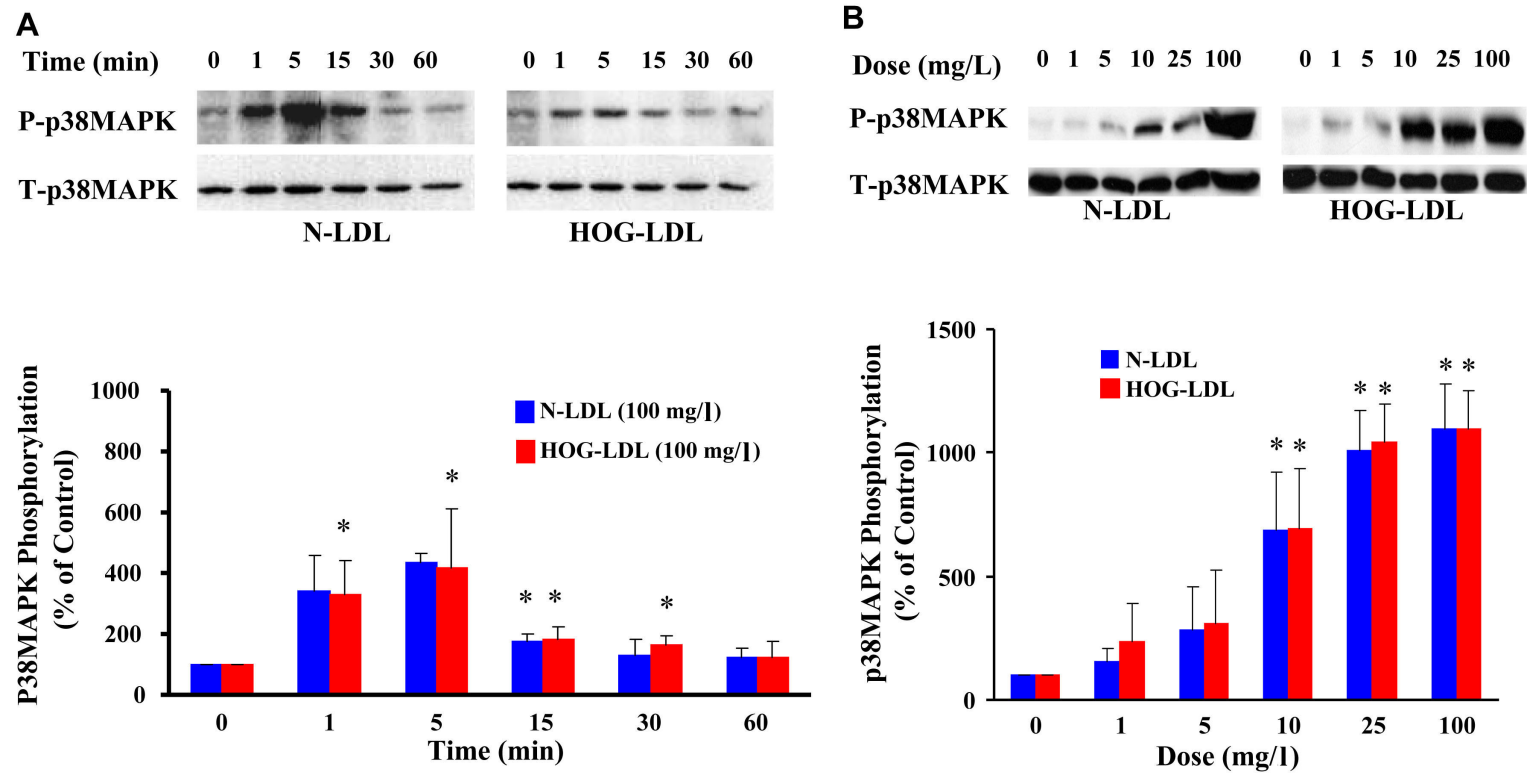
LDL increases p38MAPK phosphorylation. **A:** This panel shows representative western immunoblots from one experiment, and densitometric data from three experiments (mean±SD) describing the time course of LDL-induced p38 MAPK phosphorylation (P-p38MAPK and T-p38MAPK means phosphorylated and total p38 MAPK). **B:** This panel shows representative western immunoblots from one experiment, and densitometric data from three experiments (mean±SD) describing dose effects of LDL on p38 MAPK phosphorylation. N-LDL and HOG-LDL had similar effects on p38 MAPK phosphorylation. Control immunosignal (T-p38MAPK) bands were detected in the same gels as P-p38MAPK after stripping and re-probing. In both panels, densitometric calculations of P-p38MAPK were corrected for T-p38MAPK. Asterisk represents p<0.05 compared to control (Time 0 or Dose 0).

Quiescent HRCP were incubated with 100 mg/l of N-LDL and HOG-LDL for 1, 5, 15, 30, and 60 min. For each LDL preparation, p38 MAPK phosphorylation increased at 1 min, peaked at 5 min, and then declined (Figure 5A). Responses to N-LDL and HOG-LDL were similar.

#### Dose-dependent p38 MAPK responses at lower concentrations of N-LDL and HOG-LDL

Quiescent HRCP were incubated for 5 min with 1, 5, 10, 25, and 100 mg/l of N-LDL and HOG-LDL. N-LDL and HOG-LDL showed similar effects on p38 MAPK phosphorylation, with similar dose–response curves (Figure 5B).

#### Effect of p38 inhibition on apoptosis

**Figure 6:**
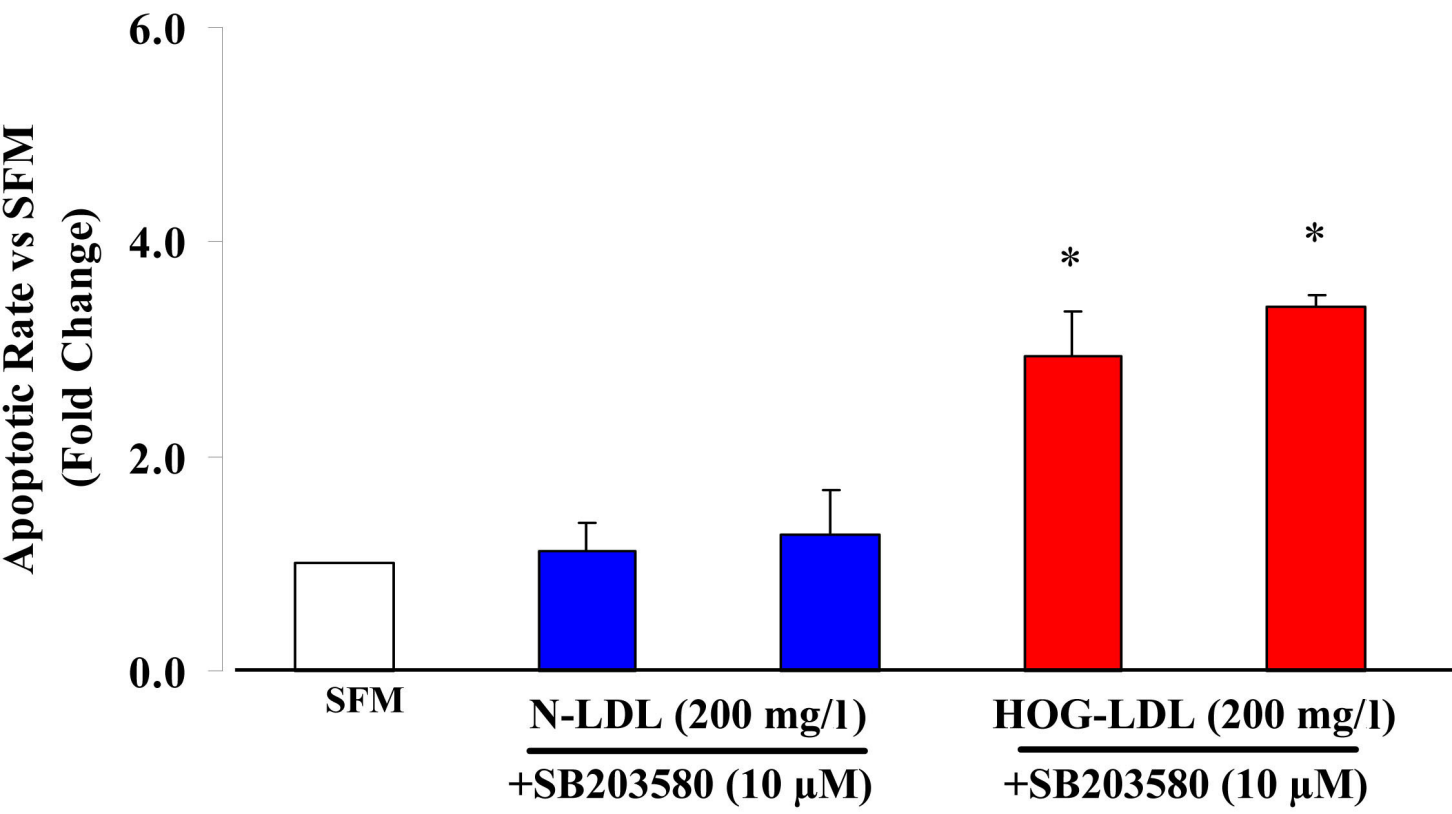
Effects of SB203580 on HOG-LDL-induced apoptosis. Administration of 10 µM SB203580, an inhibitor of p38MAPK signaling pathways, did not inhibit highly oxidized-glycated low density lipoprotein (HOG-LDL)-induced apoptosis in human retinal capillary pericytes (HRCP). Bars represent mean±SD of three separate experiments. The asterisk indicates p<0.05 compared to serum-free medium (SFM).

SB203580 was employed as an inhibitor of p38 MAPK. SB203580 (10 µM) did not inhibit apoptosis induced by HOG-LDL at 100 mg/l (data not shown) or at 200 mg/l (Figure 6).

### JNK activation

#### Phosphorylation of JNK by native and modified LDL

**Figure 7:**
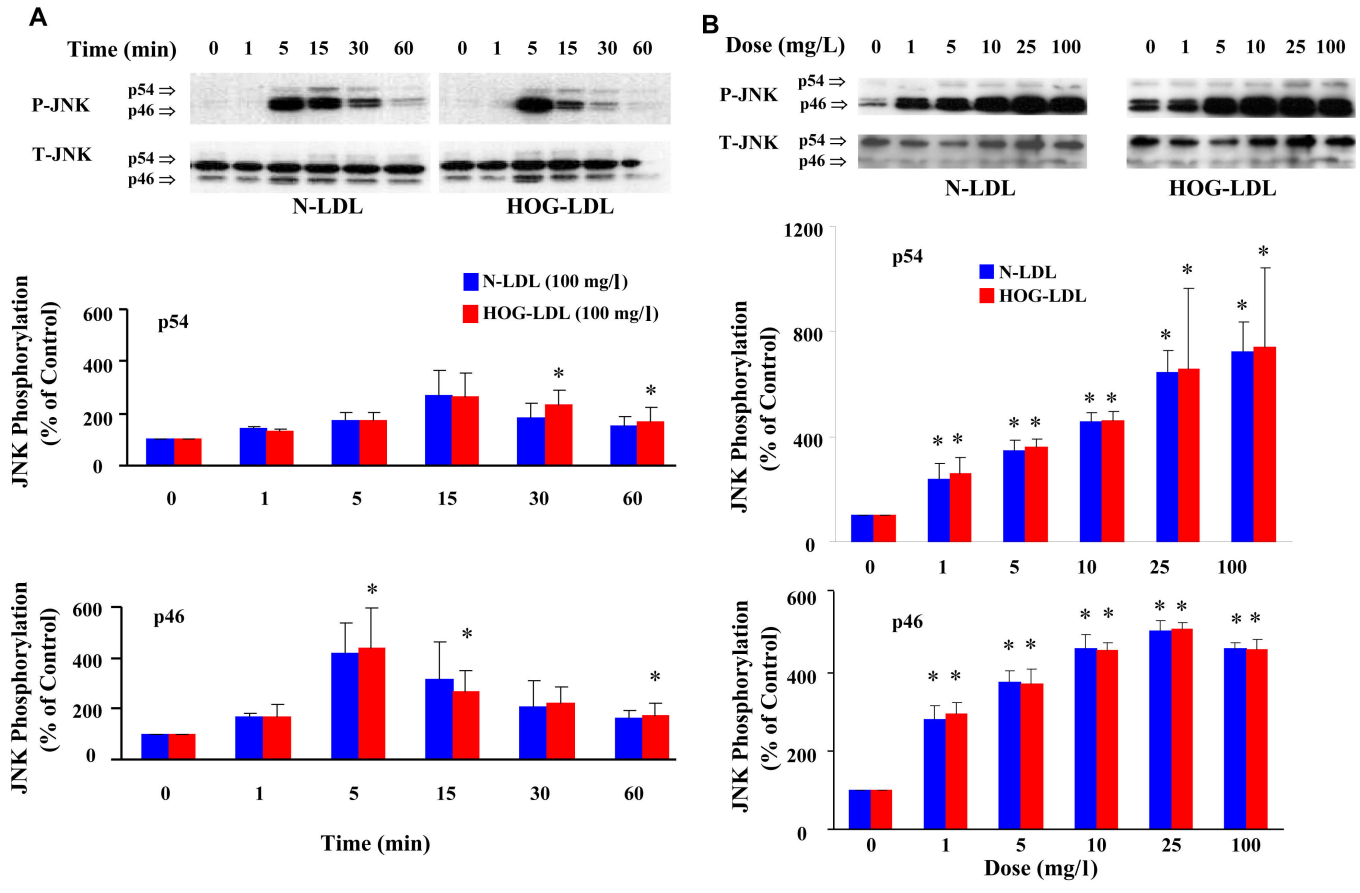
LDL increases JNK phosphorylation. **A:** This panel shows representative western immunoblots from one experiment, and densitometric data from three experiments (mean±SD) describing the time course of LDL-induced phosphorylation of the two isoforms of JNK, p54, and p46 (P-JNK and T-JNK means phosphorylated and total JNK). **B:** This panel shows representative western immunoblots from one experiment, and densitometric data from three experiments (mean±SD) describing dose effects of LDL on p54 and p46 JNK phosphorylation. N-LDL and HOG-LDL had similar effects on phosphorylation of both JNK isoforms. Control immunosignal (T-JNK) bands (both isoforms) were detected in the same gels as P-JNK after stripping and re-probing. In both panels, densitometric calculations of P-JNK were corrected for T-JNK. Asterisk represents p<0.05 compared to control (Time 0 or Dose 0).

Quiescent HRCP were incubated with 100 mg/l of N-LDL and HOG-LDL for 1, 5, 15, 30, and 60 min. JNK p46 phosphorylation was increased at 1 min, peaked at 5 min, and then declined. JNK p54 phosphorylation peaked at 15 min (Figure 7A). N-LDL and HOG-LDL showed similar effects on JNK phosphorylation.

#### Dose-dependent JNK response at lower concentrations of N-LDL and HOG-LDL

Quiescent HRCP were incubated for 5 min with 1, 5, 10, 25, and 100 mg/l of N-LDL, and HOG-LDL. N-LDL and HOG-LDL exhibited similar dose-dependent response curves for phosphorylation of JNK (Figure 7B).

#### Effect of JNK inhibition on apoptosis

**Figure 8:**
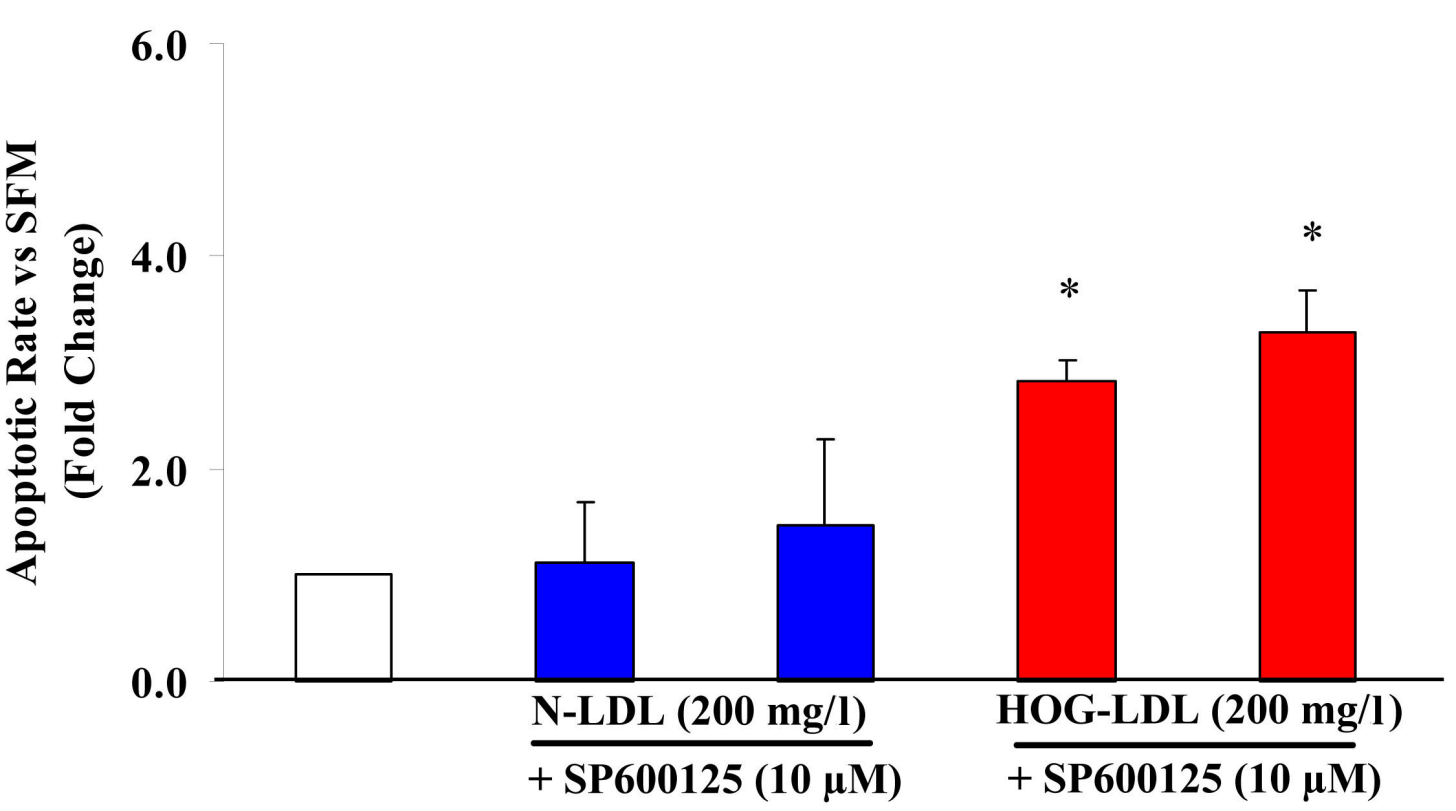
Effects of SP600125 on HOG-LDL-induced apoptosis. Administration of 10 µM SP600125, an inhibitor of Jun N-terminal kinase (JNK) signaling pathways, did not block apoptosis in human retinal capillary pericytes (HRCP) induced by highly oxidized-glycated low density lipoprotein (HOG-LDL). Bars represent mean±SD of three separate experiments. The asterisk represents p<0.05 compared to serum-free medium (SFM).

SP600125 was employed as an inhibitor of JNK. SP600125 (10 µM) did not inhibit apoptosis induced by HOG-LDL at 100 mg/l (data not shown) or at 200 mg/l (Figure 8).

To block MAPK signaling pathways, we used specific inhibitors U0126 (ERK), SB203580 (p38), and SP600125 (JNK) [24-26]. None inhibited apoptosis triggered by HOG-LDL. At 10 μM, each inhibitor is known to block activation of its target enzyme [27-29]. In separate experiments, we confirmed their efficacy: HRCP were pre-incubated with 10 μM of each inhibitor for 1 h, then co-exposed to inhibitors and HOG-LDL for 30 or 60 min, and significant reductions in pathway activity were observed (data not shown).

## Discussion

Retinal capillary pericyte loss by apoptosis is an established early feature of DR. Pericytes are numerous in retinal capillaries compared to other capillary beds. They regulate endothelial cell proliferation and survival, and maintain the integrity of retinal capillaries. In DR, they undergo apoptosis that results in so-called “pericyte ghosts.” Dyslipidemia, defined as qualitative and quantitative abnormalities of plasma lipoproteins, is associated with the severity of DR based on accumulating evidence from clinical studies including the Diabetes Control and Complications Trial/Epidemiology of Diabetes Intervention and Complications (DCCT/EDIC), Hoorn, and Pittsburgh Epidemiology of Diabetic Complications studies [30-32]. For example, we found associations between DR and adverse plasma lipoprotein subclass distributions, especially in men [31]. Retinal hard exudates regress significantly after correction of dyslipidemia [33,34]. However, we hypothesize that the most important effects of plasma lipoproteins in the promotion of DR occur after they are extravasated through a damaged blood retinal barrier, become modified, and mediate injury to cells in their vicinity.

Oxidation of LDL in the subintimal space of arterial walls is now accepted as a potent stimulus for atherogenesis [35-37]. In the present and related work, we hypothesize that an analogous process is implicated in DR [14,15,17,18]. This notion is strengthened by the demonstration of extravasated LDL, using immunohistochemistry, in wet macular edema [33], and by ourselves in human retinal samples from diabetic and normal controls [16]. Our studies demonstrated the presence of oxidized and aggregated LDL in human retina in diabetics in proportion to the severity of DR [16]. In contrast, oxidized LDL is absent in the absence of diabetes. These findings are consistent with potential roles of modified LDL in the initiation and development of DR. Furthermore, we have shown that HOG-LDL has strong cytotoxic effects on cultured bovine retinal pericytes and endothelial cells in vitro [14] and induces the programmed cell death of human pericytes [15,16]. In the present study we investigate the involvement of the three MAPK subfamilies–ERK, JNK, and p38–in apoptosis induced by HOG-LDL versus N-LDL in HRCP.

MAPKs have been shown to be the central mediators that propagate extracellular signal inputs from cell membrane to the nucleus. They have been shown to play a key role in apoptosis in different tumor cell lines [38-40] and in primary cell cultures [41,42]. At least three structurally related MAPK subfamilies have been identified in mammalian cells: ERK, p38, and JNK. MAPK subfamilies are activated in response to different extracellular stimuli, have different downstream targets, and therefore perform different functions including mediation of apoptosis, proliferation, angiogenesis, and inflammation [43-45].

It is generally accepted that ERK activation plays a principal role in cell proliferation, differentiation, and apoptosis [43,46]. In the present study, we show that N-LDL and HOG-LDL induced a rapid phosphorylation of ERK in retinal capillary pericytes. We demonstrate that N-LDL and HOG-LDL showed the same time course of ERK activation, peaking at 5 min. Our data are in general agreement with previous studies that showed native and copper-oxidized LDL stimulated ERK1/2 activation in other cells such as renal mesangial cells or vascular smooth muscle cells (VSMC) [23,47]. However, the relative effects of the different LDL preparations vary among the various vascular cell types. HOG-LDL is a more potent activator of ERK than N-LDL in renal mesangial cells [23], while N-LDL induces a more potent response than HOG-LDL in VSMC [47]. In contrast to those studies, N-LDL and HOG-LDL induced similar increases in ERK1/2 phosphorylation in HRCP. The sensitivity of vascular cells to normal and modified LDL also seems to vary between cell types. In renal mesangial cells, we found dose-dependent ERK activation in doses up to 100 mg/l. We also demonstrated that at a low dose (10 mg/l), the effect of N-LDL was greater than that of HOG-LDL [23]. Our findings with retinal pericytes confirm the dose-dependency of the ERK response but showed that it occurs at lower concentrations (maximal at 10–25 mg/l) than in renal mesangial cells (maximal at 50–100 mg/l) and to be detectable at very low LDL concentrations (1 and 5 mg/l). This indicated that relatively small amounts of N-LDL and modified LDL may trigger retinal cell signaling cascades in pericytes, which may influence retinal responses to diabetes.

It has been established that p38 MAPK can be activated by a variety of environmental stresses such as osmotic shock, ultraviolet radiation, heat shock, and pro-inflammatory cytokines, and that activation of p38 MAPK cascades can trigger apoptosis [48]. Concentration- and time-dependent activation of p38 MAPK by oxidized LDL in rat VSMC (maximal at 100 mg/l within 5 min) was demonstrated by Jing et al. [49]. In contrast, N-LDL was a much weaker activator of this pathway. Our present data show that N-LDL and HOG-LDL induced p38 MAPK phosphorylation in a time-dependent manner in human retinal pericytes, peaking at 5 min, but the responses to the two lipoprotein preparations were similar.

The JNK family of protein kinases, also known as SAPK, is implicated in apoptosis [50-52]. The JNK family includes three genes, JNK1, JNK2, and JNK3, each of which can produce 46 kDa and 54 kDa isoforms [53,54]. Zhu et al. found that N-LDL activated JNK in human endothelial cells, starting at 15 min and peaking at 1–2 h [55]. In the present study, p46JNK phosphorylation was increased at 1 min, peaked at 5 min, and then declined. p54 JNK phosphorylation peaked at 15 min. As with ERK and p38 MAPK, there was no difference in the magnitude of responses to N-LDL and HOG-LDL.

Inhibition of each of the three MAPK pathways had no effect in preventing apoptosis following exposure to HOG-LDL. All of our data therefore suggest that these MAPK pathways do not play a significant role in HOG-LDL-induced apoptosis in HRCP, and that the responses in these cells differ both qualitatively and quantitatively from those studied from the kidney (renal mesangial cells) and arterial wall (VSMC). Although HOG-LDL-induced apoptosis did not depend on altered MAPK pathway activation, several studies have suggested that MAPK pathways are implicated in pathogenesis of DR. MAPK pathways are recognized as mediators of cellular responses to elevated glucose levels, and hence as mediators of the development of the complications of diabetes, including DR [56]. MAPK has been shown to respond to several forms of cellular stress that are present in DR and to be associated with biochemical abnormalities relevant to the progression of DR [57,58]. Examples are as follows: prevention and reversal of diabetic retinal vascular changes by angiopoietin-1 was attributed in part to decreased MAPK activity [59]; inhibition of endothelial cell growth by PEDF may result from inhibition of vascular endothelial growth factor (VEGF)-induced MAPK activation [60]; and inhibition of VEGF-induced angiogenesis by the hepatocyte growth factor/NK4 depends on inhibition of ERK activation in both in vitro and in vivo models [61]. Therefore inhibitors of MAPK pathways may have potential uses in attenuating VEGF-induced angiogenesis and revascularization [62].

Besides apoptosis, MAPK signaling pathways mediate inflammation (e.g., by stimulating arachidonic acid synthesis [44]), angiogenesis by promoting formation of capillary-like structures in co-cultured human umbilical vein endothelial cells and fibroblasts [45], and atherosclerosis by activating scavenger receptors, vascular smooth muscle proliferation and forming foam cells [47,63,64]. Furthermore, our previous studies showed that when cells are exposed to HRCP to HOG-LDL, then compared to cells exposed to N-LDL or SFM, there is an alteration in specific genes including several implicated in fatty acid and eicosanoid metabolism, fibrinolytic regulation, tissue inhibition of metalloproteinases, and angiogenesis [15,18]. Therefore, activation of MAPK signaling pathways by HOG-LDL and N-LDL in HRCP might be implicated in other pathways of retinal injury, e.g., involving inflammation [44] and angiogenesis [65,66]. Future studies are necessary to elucidate these potential roles of HOG-LDL and the different effects of N-LDL and HOG-LDL in DR. It must be borne in mind that under normal circumstances, neither version of the lipoprotein leaks into the retinal extravascular space. Another direction for future work is to determine molecular signaling pathways stimulated by multiple exposures of HOG-LDL, which might mimic repetitive stresses under biologic conditions.

With regard to potential alternative mechanisms by which HOG-LDL elicits apoptosis in retinal pericytes, we have demonstrated that HOG-LDL increased Bax protein level [16], a well known marker for mitochondrial dysfunction. Further, caspases 3 and 7 were activated after 24 h incubation of HRCP with HOG-LDL [16]. Activation of cyclooxygenase-2 pathways and enhanced oxidative stress are also implicated in pericyte loss triggered by HOG-LDL [67].

The LDL concentration range was chosen as representative of LDL concentrations that pericytes may be exposed to in vivo in diabetes. For example, a concentration of 100 mg/l of LDL protein corresponds to 10 to 20% of the normal circulating level of LDL. It is estimated that between 5 to 25% of circulating LDL may be non-enzymatically modified in vivo in diabetes [18].

In summary, we report that HOG-LDL induced apoptosis in cultured HRCP in a time- and dose-dependent manner. All three MAPK component pathways in retinal capillary pericytes were activated by both native and modified LDL, all in a time- and dose-responsive manner. However, responses to N-LDL and HOG-LDL were similar, and blockade of individual pathways for ERK, p38, and JNK did not prevent HOG-LDL-induced apoptosis. Therefore, although they may mediate other responses of pericytes in DR, MAPK pathways do not appear to be directly involved in the induction of apoptosis in these cells.
